# Two new species of the orb-weaver genus *Chorizopes* from Yunnan, China (Araneae, Araneidae)

**DOI:** 10.3897/zookeys.626.7485

**Published:** 2016-10-20

**Authors:** Xiao-Qi Mi, Cheng Wang, Xian-Jin Peng

**Affiliations:** 1 College of Biological, Agricultural and Forest Engineering, Tongren University, Tongren, Guizhou 554300, China; 2 College of Life Sciences, Hunan Normal University, Changsha, Hunan 410081, China

**Keywords:** Diagnosis, spider, taxonomy

## Abstract

Two new species of the orb-weaver genus *Chorizopes* from Yunnan Province, China are described: *Chorizopes albus* **sp. n**. (male and female) from the Gaoligong Mountains and Ailao Mountains, and *Chorizopes longus* **sp. n.** (male and female) from the Gaoligong Mountains. *Chorizopes albus* **sp. n**. can be distinguished from the related species *Chorizopes shimenensis* by: 1) median apophysis widest at the middle part versus widest at the base in the latter; 2) median apophysis without the dorsal spur found in that of the latter; 3) spermathecae spherical versus ovoid in the latter; 4) having one pair of large white spots on posterior lateral area of abdomen versus having two pairs of crescent white patches with dark edges on dorsal abdomen in the latter. *Chorizopes longus* **sp. n**. can be separated from the similar species *Chorizopes tumens* by: 1) the median apophysis having a knob on the distal half versus having a knob on the basic half in the latter; 2) male palp having a spur versus absent in the latter; 3) the width of the groove between the paracymbium and cymbium as thick as the paracymbium versus two times as thick as the paracymbium in the latter; 4) copulatory duct attached on anterior ventral of the spermatheca versus on anterior dorsal in the latter. Photos of body and copulatory organs, line drawings of copulatory organs, as well as the distribution data are provided. The type specimens are deposited in the College of Life Sciences, at the Hunan Normal University
(HNU) and the Museum of Tongren University (MTU).

Hunan Normal University

Museum of Tongren University

## Introduction

The orb-weaver spider genus *Chorizopes* is characterised by an extremely elevated cephalic region and wide separation of lateral eyes from median eyes. At present, 25 species from Asia and Africa are included in this genus (World Spider Catalog 2016). Of these, eight have been reported to come from China (Schenkel 1963, Yin et al. 1990, Yin, Peng and Wang 1994, Yin, Wang and Xie 1994, Yin et al. 1997, Yin et al. 2012; Zhu et al. 1994, Song et al. 1999, Zhu and Zhang 2011). Among the eight known Chinese species, *Chorizopes khanjanes* and *Chorizopes wulingensis* have the following common characters: dorsum of opisthosoma having three pairs of lateral tubercles and three vertically arranged caudal tubercles, epigynum having a short scape, teeth of chelicerae serrated and loosely arranged. The remaining six species share the following characters: dorsum of opisthosoma having one pair of lateral tubercles and two vertically arranged posterior tubercles, scape absent, chelicerae teeth tightly arranged.

While examining specimens collected in Yunnan Province, in southwest China, two new species belonging to the genus *Chorizopes* were identified and are described in this paper.

## Material and methods

Specimens are kept in 75% ethanol. The epigynum was cleared in lactic acid for examination. An Olympus SZX16 stereo microscope was used for specimen examination. Digital photographs were taken using a Canon Powershot G12 digital camera mounted on an Olympus SZX16. Compound focus images were generated using Helicon Focus software. Leg measurements are given as: total length (femur, patella + tibia, metatarsus, tarsus). All measurements are given in millimetres (mm).

### Abbreviations

ALE anterior lateral eyes;

AME anterior median eyes;

C conductor;

CD copulatory ducts;

CO copulatory openings;

E embolus;

FD fertilization ducts;

HNU
Hunan Normal University;

MA median apophysis;

MOA median ocular area;

MTU Museum of Tongren University;

PLE posterior lateral eyes;

PME posterior median eyes;

S spermatheca;

TA terminal apophysis.

## Taxonomy

### Family Araneidae Clerck, 1757

#### Genus Chorizopes

Taxon classificationAnimaliaAraneaeAraneidae

O. P.-Cambridge, 1870

##### Type species.

*Chorizopes frontalis* O. Pickard-Cambridge, 1870

#### Chorizopes albus sp. n.

Taxon classificationAnimaliaAraneaeAraneidae

http://zoobank.org/6FAA3F69-6B44-44C9-A804-9115CE107A5E

Figs 1–2 , 3–4 , 5–8 , 9–12

##### Type material.

**Holotype**: male, China: Yunnan Province, Jingdong County, Huashan Township, Wengang Village, 24.3389°N, 101.1410°E, 1728 m, 16 August 2015, Cheng Wang, Zhaolin Liao, Peng Luo and Gaotao Liu leg (MTU-WC20150816). Paratypes: 1 male and 2 females, same data as holotype (MTU-WC20150816); 1 male and 2 females, Yunnan Province, Jingdong County, Huashan Township, Yingpan Village, 24.2788°N, 101.0979°E, 1273 m, 15 August 2015, Cheng Wang, Zhaolin Liao, Peng Luo and Gaotao Liu leg (MTU-WC20150815); 1 female, Yunnan Province, Fugong County, Shangpa Township, 26.8620°N, 98.8714°E, 1177 m, 19–27 August 2005, Tang Guo leg (HNU-Tang0509).

##### Etymology.

The specific name comes from the Latin *albus*, meaning whitish, referring to the large white spots on lateral abdomen; adjective.

##### Diagnosis.

The new species can be distinguished from all known congeneric species by the presence of a pair of white spots on lateral abdomen (Figs 1–2), median apophysis C-shaped and widest at the middle part (Figs 5, 9), copulatory ducts short and twisted between the spermathecae, and the epigastric furrow (Figs 7–8, 11–12).

**Figures 1–2.**
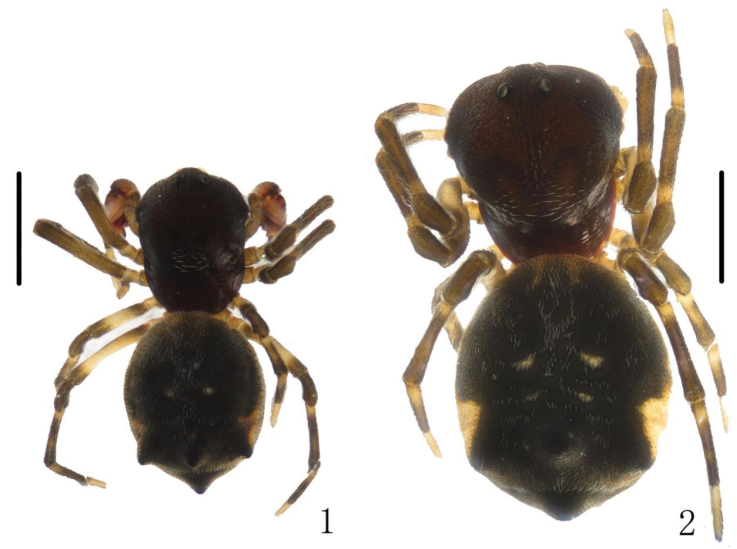
*Chorizopes albus* sp. n. **1** male habitus, dorsal view **2** female habitus, dorsal view. Scale bars 1 mm.

**Figures 3–4.**
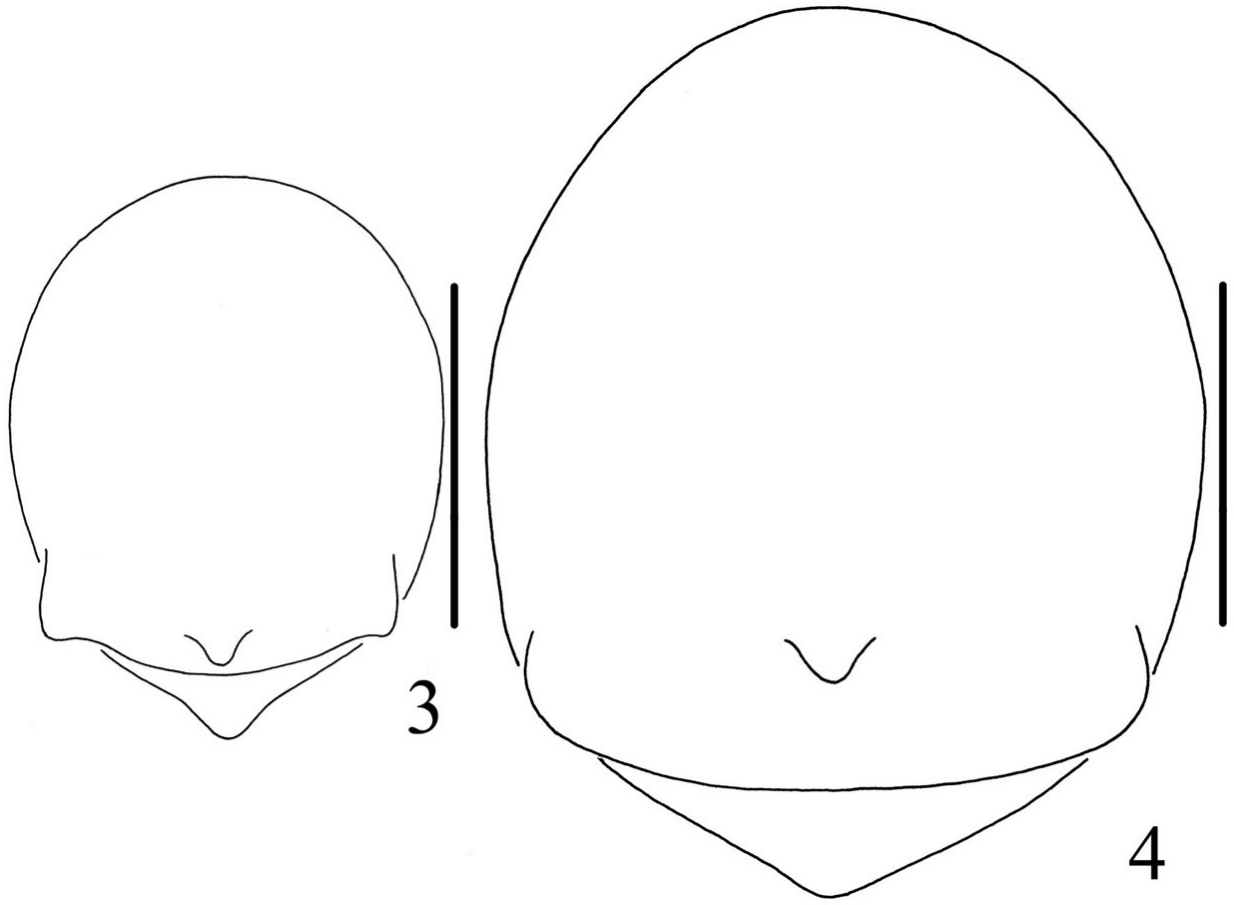
*Chorizopes albus* sp. n. **3** male abdomen, dorsal view **4** female abdomen, dorsal view. Scale bars 1 mm.

**Figures 5–8.**
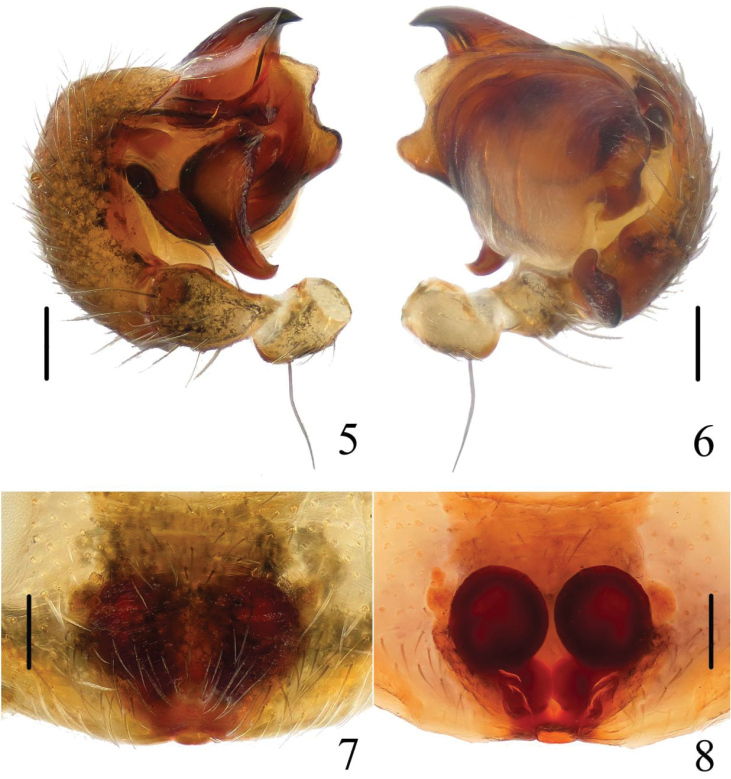
*Chorizopes albus* sp. n. **5** left palp, prolateral view **6** left palp, retrolateral view **7** epigynum, ventral view **8** vulva, dorsal view. Scale bars 0.1 mm.

**Figures 9–12.**
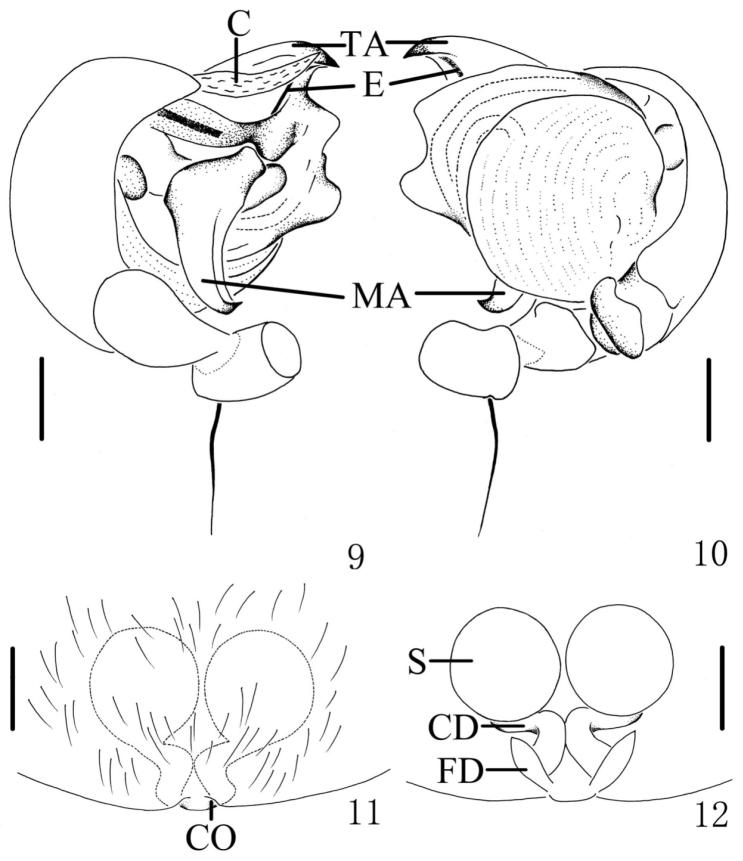
*Chorizopes albus* sp. n. **9** left palp, prolateral view **10** left palp, retrolateral view **11** epigynum, ventral view **12** vulva, dorsal view. Scale bars 0.1 mm.

##### Description.

**Male** (holotype): Carapace dark brown, hairy, elevated in cephalic region. Chelicerae dark brown, have seven promarginal teeth. Sternum triangular, pointed posteriorly, yellowish brown. Legs yellowish brown, with wide darker grey annuli. Abdomen with one pair of lateral tubercles and two vertically arranged caudal tubercles. Dorsal abdomen greyish with two white spots, ventral yellowish brown, one pair of big white spots situated on the posterior lateral (Figs 1, 3). Spinnerets yellowish brown, palp with one patellar bristle; paracymbium flattened, basally located; median apophysis prominent, widest at the middle part, with a spur at distal end; membranous conductor narrow and long, guiding the embolus; embolus slender and twisted; terminal apophysis large, pointed distally (Figs 5–6, 9–10). Total length 2.90. Carapace length 1.25, width 1.00; abdomen length 1.65, width 1.30. Eye sizes and interdistances: AME 0.13, ALE 0.08, PME 0.10, PLE 0.08, AME–AME 0.10, AME–ALE 0.48, PME–PME 0.20, PME–PLE 0.50, MOA length 0.30 with front width 0.30 and back width 0.35. Leg measurements: I 2.95 (0.90, 1.05, 0.60, 0.40), II 2.90 (0.90, 1.00, 0.60, 0.40), III 1.90 (0.60, 0.65, 0.35, 0.30), IV 2.85 (0.90, 1.05, 0.55, 0.35).

**Female** (based on one of WC20150816): Colouration and body shape same as in male (Figs 2, 4). Epigynum is a slightly convex plate, copulatory openings posteriorly situated; copulatory ducts thick and twisted; spermathecae spherical and almost touched (Figs 7–8, 11–12). Total length 4.05. Carapace length 1.50, width 1.30; abdomen length 2.60, width 1.56. Eye sizes and interdistances: AME 0.15, ALE 0.08, PME 0.10, PLE 0.08, AME–AME 0.13, AME–ALE 0.60, PME–PME 0.25, PME–PLE 0.65, MOA length 0.35 with front width 0.35 and back width 0.43. Leg measurements: I 2.80 (0.85, 1.00, 0.55, 0.40), II 2.85 (0.90, 1.00, 0.55, 0.40), III 2.15 (0.65, 0.75, 0.40, 0.35), IV 3.15 (1.05, 1.10, 0.60, 0.40).

##### Variation.

Males, total length 2.65–2.90, females, total length 3.35–4.05.

##### Distribution.

China (Yunnan Province).

#### Chorizopes longus sp. n.

Taxon classificationAnimaliaAraneaeAraneidae

http://zoobank.org/D1E2E0F8-0881-445F-9DFC-23D772FE391D

Figs 13–14 , 15–16 , 17–20 , 21–24

##### Etymology.

The specific name comes from the Latin *longus*, meaning long, referring to the long median apophysis; adjective.

##### Diagnosis.

The new species can be separated from all known congeneric species by: the median apophysis extremely long, more than 2/5 portions beyond the edge of the genital bulb in prolateral view (Figs 17–18, 21–22), and having a spur near the base (arrowed in Figs 17, 21); the copulatory ducts long and attaching the spermathecae at the anterior ventral surface (Figs 19–20, 23–24).

**Figures 13–14.**
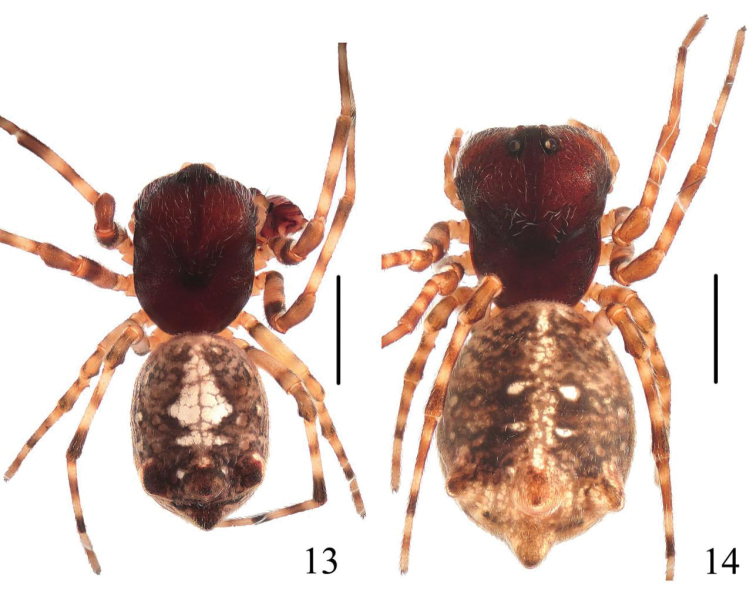
*Chorizopes longus* sp. n. **13** male habitus, dorsal view **14** female habitus, dorsal view. Scale bars 1 mm.

**Figures 15–16.**
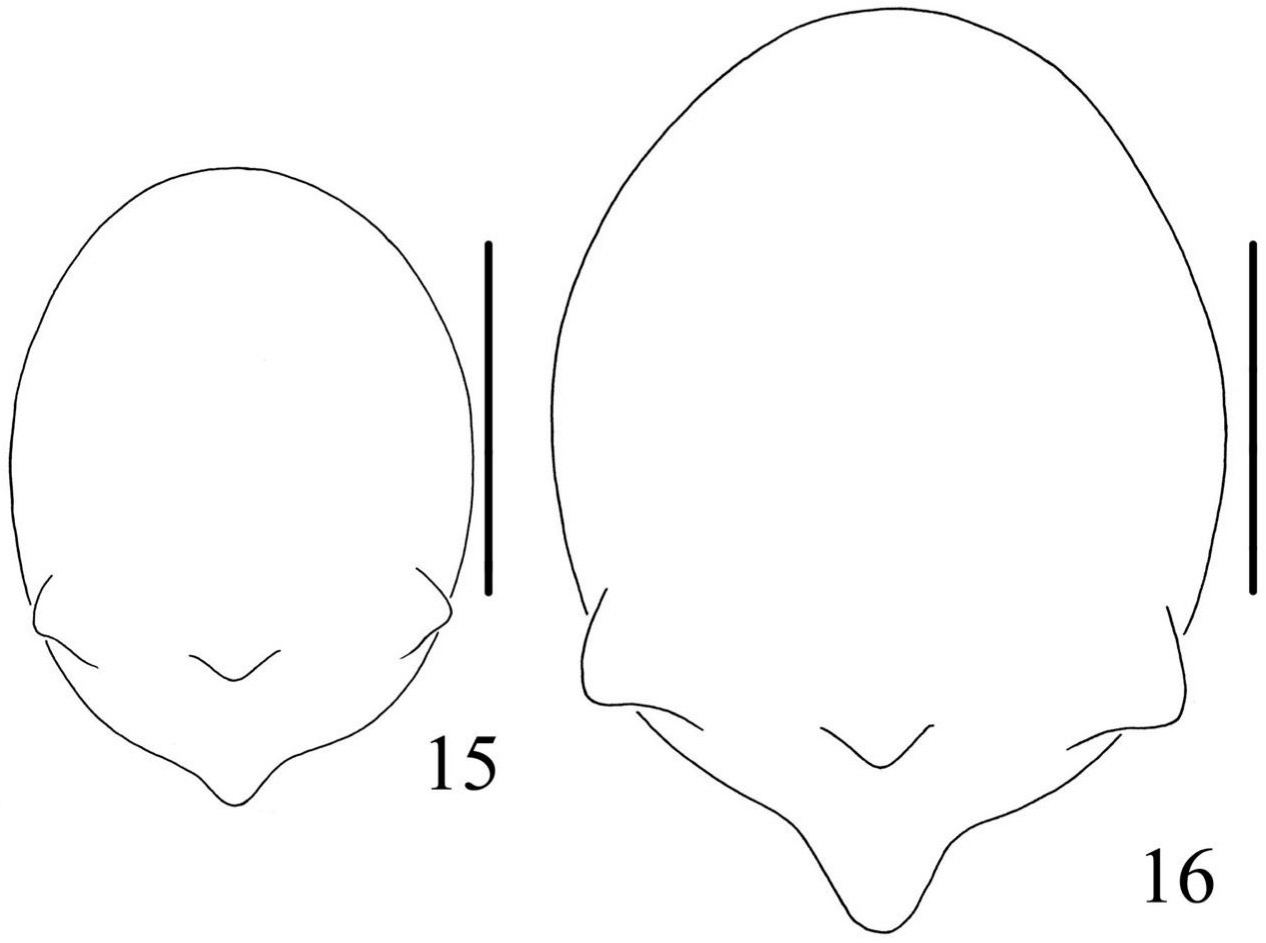
*Chorizopes longus* sp. n. **15** male abdomen, dorsal view **16** female abdomen, dorsal view. Scale bars 1 mm.

**Figures 17–20.**
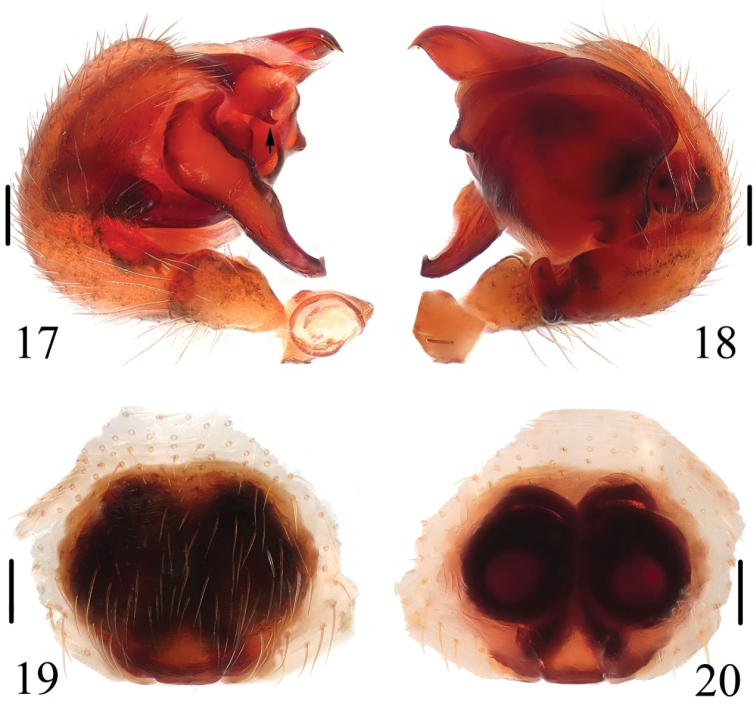
*Chorizopes longus* sp. n. **17** left palp, prolateral view **18** left palp, retrolateral view **19** epigynum, ventral view **20** vulva, dorsal view. Scale bars 0.1 mm.

**Figures 21–24.**
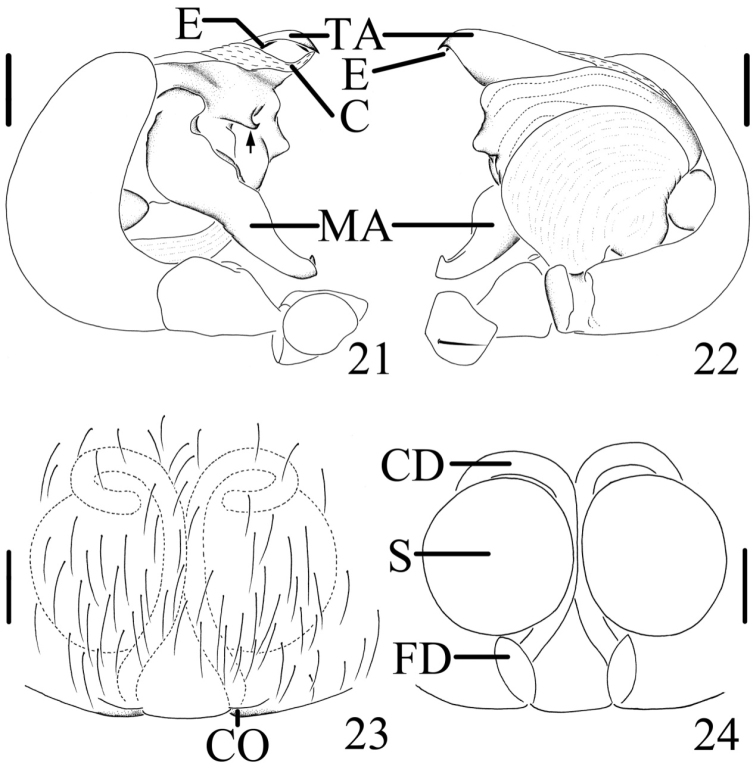
*Chorizopes longus* sp. n. **21** left palp, prolateral view **22** left palp, retrolateral view **23** epigynum, ventral view **24** vulva, dorsal view. Scale bars 0.1 mm.

##### Type materials.

Holotype, male, CHINA, Yunnan Province, Tengchong County, Houqiao Township, Yangjiatian Village, 25.3539°N, 98.2549°E, 1785 m, 28 May 2006, Xinping Wang and Peng Hu (HNU-WH060528). Paratypes: 1 male and 1 female, the same data as holotype (HNU-WH060528); 2 females, CHINA, Yunnan Province, Gongshan County, Bingzhongluo Township, Bingzhongluo Village, 30.7 air km NNW of Gongshan, in a small cave, 28.0194°N, 98.6211°E, 1760 m, 7-8 July 2000, Hengmei Yan, David Kavanaugh, Charles Griswold, Hongbin Liang, Darrell Ubick, & Dazhi Dong (HNU-00-GBC); 1 female, CHINA, Yunnan Province, Fugong County, Pihe Township, Wawa Village, 26.5903°N, 98.9082°E, 1263 m, 13 May 2004, Hengmei Yan (HNU-20040513); 1 female, CHINA, Yunnan Province, Tengchong County, Mingguang Township, Zizhi Village, Cizhuhe, 25.4560°N, 98.3703°E, 2120 m, 21 May 2006, Changmin Yin & Jiafang Hu (HNU-YHY09); 2 male and 2 female, CHINA, Yunnan Province, Tengchong Couty, Jietou Township Shaba Village, 25.3926°N, 98.7034°E, 1850 m, 25 May 2006, Xinping Wang and Peng Hu (HNU-WH060525); 1 female, CHINA, Yunnan Province, Tengchong Couty, Houqiao Township, Doujiazhai Village, 25.3578°N, 98.2274°E, 1673 m, 30 May 2006, Xinping Wang and Peng Hu (HNU-WH060530).

##### Description.

**Male** (holotype): Carapace dark brown, hairy, cephalic region elevated. Chelicerae dark brown, have seven promarginal teeth. Sternum triangular, dark brown. Gnathocoxae and labium yellowish brown. Legs yellowish brown with dark grey annuli. Dorsal abdomen greyish brown, cardiac pattern pale and long bar-shaped, two pairs of white spots on posterior lateral of cardiac pattern, posterior area of abdomen with one pair of lateral tubercles and two vertically arranged caudal tubercles (Figs 13, 15), ventral greyish brown with white scaly patches. Spinnerets brown. Palp with one patellar bristle; paracymbium flattened, basally located; median apophysis prominent, with a knob on the distal part; membranous conductor long and narrow; embolus slender and twisted; terminal apophysis large, pointed distally (Figs 17–18, 21–22). Total length 3.40. Carapace length 1.60, width 1.25; abdomen length 1.80, width 1.35. Eye sizes and interdistances: AME 0.10, ALE 0.08, PME 0.10, PLE 0.10, AME–AME 0.10, AME–ALE 0.50, PME–PME 0.20, PME–PLE 0.55, MOA length 0.30 with front width 0.30 and back width 0.38. Leg measurements: I 4.25 (1.30, 1.35, 1.00, 0.60), II 4.30 (1.50, 1.40, 0.85, 0.55), III 2.45 (0.80, 0.80, 0.45, 0.40), IV 3.85 (1.25, 1.20, 0.90, 0.50).

**Female** (based on WH060528): Coloration and body shape same as in male (Figs 14, 16). Chelicerae have seven promarginal teeth. Epigynum has a slightly convex plate; copulatory openings posteriorly situated; copulatory ducts very long and twisted, attach the anterior ventral of the spermathecae; spermathecae big and spherical (Figs 19–20, 23–24). Total length 4.10. Carapace length 1.80, width 1.45; abdomen length 2.60, width 1.90. Eye sizes and interdistances: AME 0.13, ALE 0.10, PME 0.13, PLE 0.13, AME–AME 0.13, AME–ALE 0.75, PME–PME 0.23, PME–PLE 0.80, MOA length 0.38 with front width 0.33 and back width 0.40. Leg measurements: I 3.55 (1.00, 1.10, 0.90, 0.55), II 4.00 (1.15, 1.30, 1.00, 0.55), III 2.25 (0.75, 0.80, 0.50, 0.50), IV 3.10 (1.25, 1.40, 0.95, 0.50).

##### Variation.

Males, total length 3.10–3.40 (n=4), females, total length 3.80–4.75 (n=6).

##### Distribution.

China (Yunnan Province).

## Supplementary Material

XML Treatment for Chorizopes

XML Treatment for Chorizopes albus

XML Treatment for Chorizopes longus
